# Pulmonary aspergilloma due to *Aspergillus flavus* in an immunocompetent patient from Northeast Iran: A case report

**DOI:** 10.1016/j.idcr.2026.e02709

**Published:** 2026-08-04

**Authors:** Seyedeh Sabereh Mojtahedi, Hossein Zarrinfar, Mojtaba Taghizadeh Armaki

**Affiliations:** a Department of Parasitology and Mycology, School of Medicine, Mashhad University of Medical Sciences, Mashhad, Iran; b Students' Scientific Research Center, Tehran University of Medical Sciences, Tehran, Iran; c Allergy Research Center, Mashhad University of Medical Sciences, Mashhad, Iran; d Infectious Diseases and Tropical Medicine Research Center, Health Research Center, Babol University of Medical Sciences, Babol, Iran

**Keywords:** Antifungal susceptibility, Aspergillus flavus, Aspergilloma, Molecular identification, Pulmonary infection

## Abstract

A 27-year-old man presenting with cough, dyspnea, and an abnormal chest radiograph was clinically suspected of having a pulmonary aspergilloma. Histopathological examination of the resected lung tissue demonstrated a dense aggregation of branching, septate hyphae, and partial β-tubulin gene sequencing identified the isolate as *Aspergillus flavus*. Antifungal susceptibility testing showed the lowest MIC for posaconazole (0.125 μg/mL) and the highest for amphotericin B (4 μg/mL) and 5-fluorocytosine (>64 μg/mL). The patient was discharged after a 16-day hospitalization with complete clinical recovery and was prescribed oral itraconazole for continued outpatient management.

## Introduction

Aspergillus species are saprophytic molds found widely in the environment. They can cause a variety of pulmonary conditions, ranging from allergic reactions to severe invasive infections in patients with weakened immunity [1], [2], [3]*.* Globally, *A. fumigatus* is the most frequently encountered pathogen. However, in the Middle East, most reported cases are attributed to *A. flavus*, likely reflecting its greater ability to thrive in high temperatures and dry conditions [4], [5], [6]. Pulmonary aspergilloma (PA) is a form of chronic pulmonary aspergillosis (CPA). It arises when fungal hyphae, together with inflammatory cells and detritus, colonize an existing lung cavity and gradually form a distinct fungal ball [5], [7], [8]. The condition is most often seen in patients with pre-existing structural damage to the lungs, particularly those with old tuberculous cavities, though it may also occur in the setting of lung abscesses, sarcoidosis, pneumoconiosis, endemic mycoses, or cystic fibrosis [9], [10]. That said, PA is exceedingly rare in immunocompetent individuals with no previous lung pathology. Diagnosis in these situation can be challenging due to the nonspecific clinical sypyoms which can easily mistaken for a tumor or pyogenic infection [11]. We describe here a case of PA caused by *A. flavus* in an immunocompetent patient who had no known underlying lung disease. The work-up included histopathological examination, fungal culture, β-tubulin gene sequencing, and antifungal susceptibility testing. Surgical resection was performed, and the patient received antifungal therapy, achieving a favorable clinical outcome. This case demonstrates the importance of considering PA in the differential diagnosis of cavitary pulmonary lesions, even among immunocompetent individuals without overt structural lung disease. Furthermore, it illustrates the complementary role of conventional diagnostic tools like histopathology and fungal culture alongside molecular identification and antifungal susceptibility testing in providing a comprehensive diagnostic profile and guide treatment choices.

## Case report

**Fig. 1:**
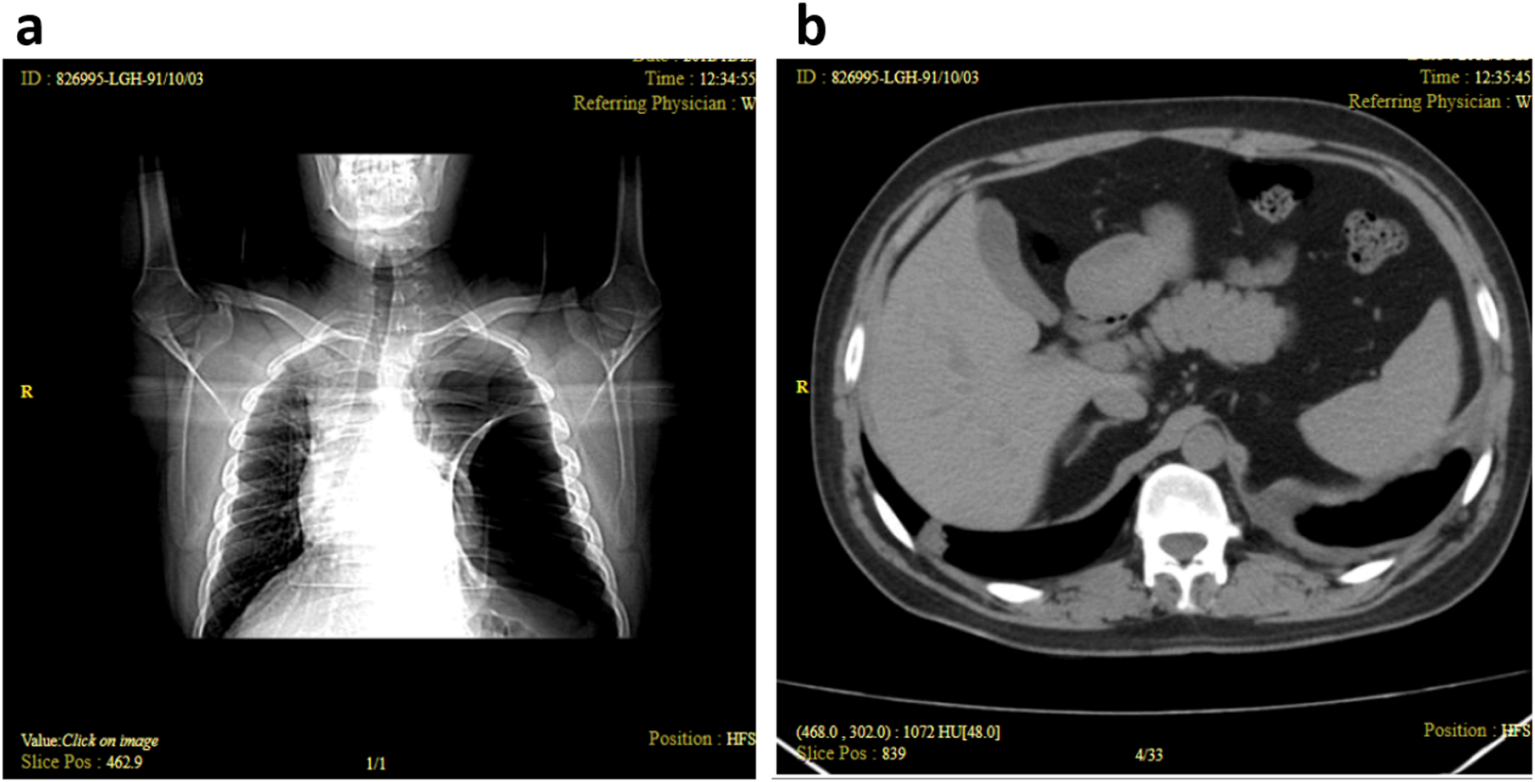
(A) Non-contrast axial chest CT scan at the level of the main bronchi showing a large left-sided pneumothorax, complete collapse of the left lung, and mediastinal shift to the right. (B) Non-contrast axial chest CT scan at the level of the left lower lobe demonstrating a thick-walled cavitary lesion containing an intracavitary soft-tissue mass (fungus ball). The surrounding crescentic rim of air (air-crescent sign) is characteristic of pulmonary aspergilloma.

A 27-year-old man from Mashhad, Iran, was admitted to the Ghaem Educational, Research, and Treatment Center (Mashhad, Northeast Iran) with persistent cough and dyspnea. Immunosuppression or extrapulmonary manifestations such as hepatic involvement or anaphylactic reactions were not observed. The cough had persisted for approximately four months, and dyspnea developed about one month after symptom onset. The patient was a non-smoker with no history of alcohol consumption or substance use. He worked as a carpenter with regular exposure to wood dust, for several years. He had no history of prior respiratory infections, including pulmonary tuberculosis. HIV serology was negative. On admission, Contrast-enhanced chest CT demonstrated a large left-sided pneumothorax with complete collapse of the left lung and mediastinal shift to the right (Fig. 1A). At the level of the left lower lobe, a cavitary lesion containing an intracavitary soft-tissue mass (fungus ball) was seen (Fig. 1B). Serum immunoglobulin levels (IgG, IgA, IgM) were within normal limits. Other laboratory findings on admission are summarized in Table 1. On the third day of admission, repeat biochemistry showed urea 37 mg/dL and creatinine 1.1 mg/dL. During hospitalization, the patient received several antibiotics, including imipenem, vancomycin, ceftriaxone, clindamycin, cefixime, and meropenem. One week after admission, laboratory tests demonstrated the following: WBC 13.8 × 10⁹/L, RBC 5.2 × 10 ¹ ²/L, Hb 14.2 g/dL, Ht 44.3%, Plt 416 × 10⁹/L, Neu 70%, Lym 25%, urea 43 mg/dL, and creatinine 1.0 mg/dL.

**Table 1:** Serial hematological and biochemical parameters of the patient on admission and during hospitalization.

Parameter	Unit	On Admission	Day 3	Day 7
WBC	× 10⁹/L	11.4	—	13.8
RBC	× 10 ¹ ²/L	4.9	—	5.2
Hemoglobin	g/dL	13.2	—	14.2
Hematocrit	%	41.3	—	44.3
Platelets	× 10⁹/L	383	—	416
Neutrophils	%	85	—	70
Lymphocytes	%	15	—	25
Blood Sugar	mg/dL	155	—	—
Urea	mg/dL	30	37	43
Creatinine	mg/dL	0.9	1.1	1.0
Sodium	mEq/L	143	—	—
Potassium	mEq/L	4.5	—	—

WBC, white blood cell count; RBC, red blood cell count; L, per liter; g/dL, grams per deciliter; mg/dL, milligrams per deciliter; mEq/L, milliequivalents per liter.

On the ninth day of admission, the patient underwent thoracic resection surgery for PA. Intraoperatively, a cavitary fungus ball was observed, and part of the left lobe was destroyed due to invasion and colonization by fungal hyphae. The adjacent pleura was also involved and thickened. Segmental resection of the left lobe, cyst removal, and visceral and parietal pleural decortication were successfully performed. Two chest tubes (28 Fr and 32 Fr) were inserted into the left hemithorax, and the pneumothorax was resolved at the end of the procedure.

**Fig. 2:**
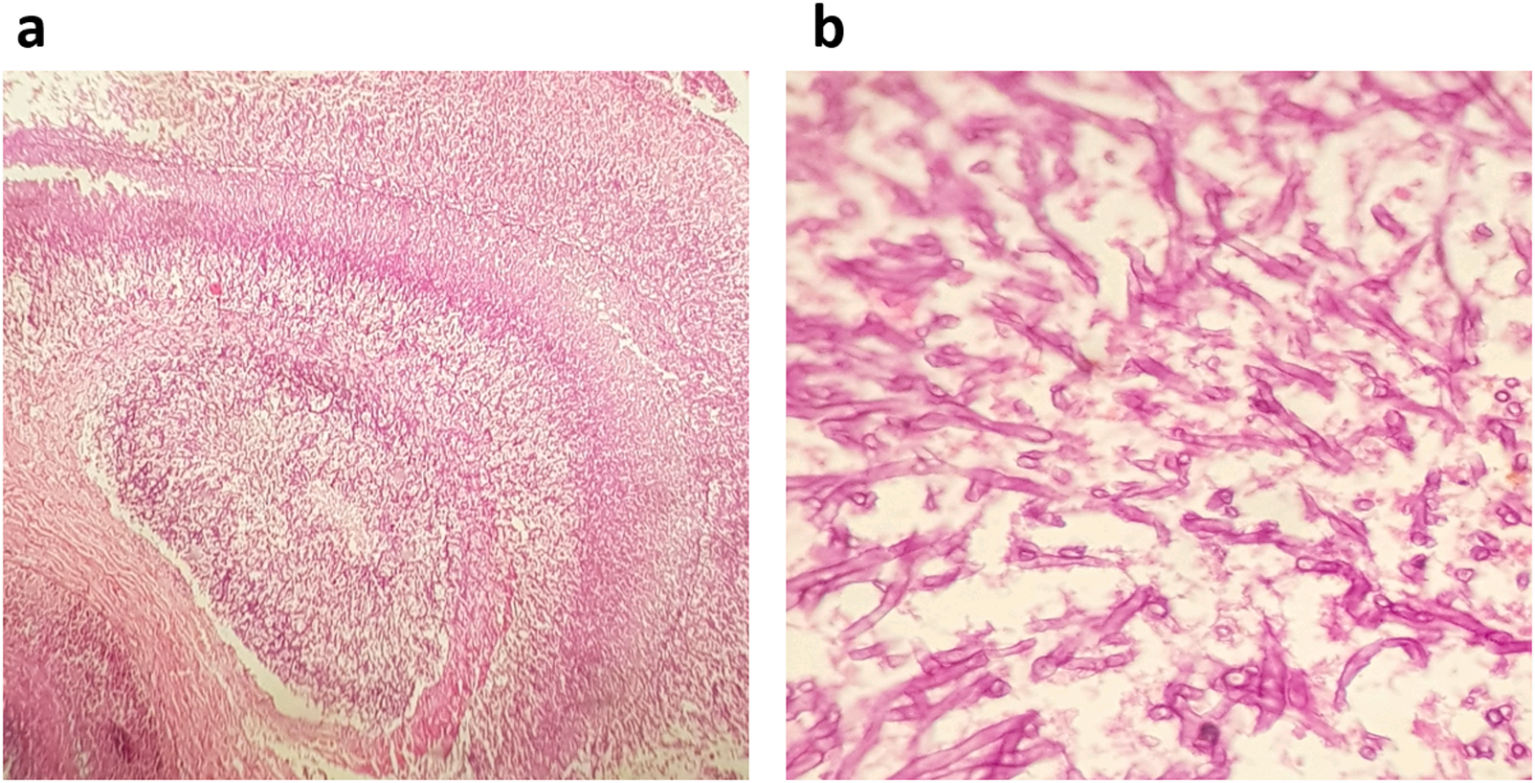
Periodic acid–Schiff (PAS)-stained sections of the resected lung tissue. (A) Low-power view demonstrating granuloma formation, infiltration of adjacent necrotic tissue, and vascular invasion. Mixed inflammatory cells surround the affected area. (B) High-power view (×400) showing dense aggregates of fungal hyphae. The hyphae are uniform, septate, and exhibit acute-angle branching (black arrowhead), consistent with *Aspergillus* species.

The resected lung specimens and portions of the involved parietal pleura were submitted to the medical mycology and pathology laboratories for further evaluation. The lung specimens consisted of two cream- to brown-colored tissue fragments measuring 1 × 3 × 6.5 cm and 1.5 × 3 × 5 cm. Sectioning revealed a cystic area containing dense yellowish to cream-colored material measuring 1 × 1.5 × 1.5 cm. The parietal pleura biopsies included a larger fragment measuring 0.5 × 5 × 12 cm with a cream-to-brown coloration and elastic consistency, and a smaller fragment measuring 1.5 × 2 × 6.5 cm with a cream-to-yellowish coloration and adipose tissue–like appearance. Histopathological examination of the lung segmentectomy specimen revealed pulmonary infarction and an abscess caused by a fungal infection morphologically consistent with *Aspergillus* species; the parietal pleura biopsy showed granulation tissue. Periodic acid–Schiff–stained sections of the resected lung demonstrated dense aggregates of fungal hyphae, granuloma formation, infiltration of adjacent necrotic tissue, and vascular invasion *(*Fig. 2*A).* The hyphae were uniform, septate, and exhibited acute-angle branching, consistent with the morphology of *Aspergillus* spp., and were surrounded by mixed inflammatory cells (Fig. 2B). Direct microscopic examination of the clinical specimen in 15% potassium hydroxide revealed septate hyphae. The biopsy was inoculated onto Sabouraud dextrose agar supplemented with chloramphenicol (SC; Merck, Germany). After three days of incubation at 35 °C, the culture produced a rapidly expanding hyaline filamentous fungal colony. Macroscopically, the colonies appeared as powdery, yellowish-green masses on the surface. Subsequently, molecular assays were performed to confirm the fungal species. Briefly, the partial β-tubulin gene was amplified by PCR in 25 µL reaction volumes containing 25 pmol of each primer (forward Bt2a: 5′-GGTAACCAAATCGGTGCTGCTTTC−3′; reverse Bt2b: 5′-ACCCTCAGTGTAGTGACCCTTGGC−3′), 2 µL template DNA, 2.5 µL 10 × reaction buffer, 1.5 mM MgCl₂, 400 µM deoxynucleotide triphosphates, 1.25 U Taq DNA polymerase, and nuclease-free water. Amplification was performed with an initial denaturation at 95 °C for 5 min, followed by 35 cycles of 94 °C for 45 s, 60 °C for 45 s, and 72 °C for 1 min, with a final extension at 72 °C for 6 min. Sequence data were edited using Lasergene SeqMan software (DNAStar, Inc., Madison, WI, USA) and analyzed using the Nucleotide Basic Local Alignment Search Tool (BLAST; http://blast.ncbi.nlm.nih.gov). The sequence was submitted to GenBank under accession number OP917826. Comparative BLAST analysis showed 99% identity with the ex-type isolate of *A. flavus*, confirming the species-level identification. In addition, in vitro antifungal susceptibility testing was performed according to the EUCAST method using eight antifungal agents: amphotericin B, itraconazole, voriconazole, posaconazole, isavuconazole, anidulafungin, caspofungin, and 5-fluorocytosine [12]. The MIC values were as follows: amphotericin B, 4 µg/mL; itraconazole, 0.25 µg/mL; voriconazole, 0.5 µg/mL; posaconazole, 0.125 µg/mL; isavuconazole, 2 µg/mL; anidulafungin, 0.032 µg/mL; caspofungin, 0.25 µg/mL; and 5-fluorocytosine, > 64 µg/mL. Following the positive culture result, the patient was initially treated with amphotericin B (0.5 mg/kg). However, due to poor tolerance and severe tremors, therapy was switched to oral itraconazole (100 mg every 12 h) after three days. The patient was discharged after 16 days of hospitalization with complete clinical improvement and continued on itraconazole therapy.

## Discussion

The PA results from the saprophytic colonization of the lungs by *Aspergillus* species and most commonly occurs in immunocompromised individuals or patients with underlying pulmonary disorders. However, PA may occasionally develop in otherwise healthy lungs, even in the absence of immunosuppression, although this presentation is extremely rare, accounting for only 0.13% of reported cases [9]. Seo et al. also emphasized that pulmonary aspergilloma in immunocompetent individuals remains uncommon despite its considerable morbidity and mortality [13]*.*

In the present case, the patient had no history of chronic pulmonary disease or immunocompromising conditions. The absence of any prior infection makes this case even more unusual. A limitation of this study is that IgG subclass testing was not performed, as this is not part of the routine diagnostic work-up at our center. However, total serum IgG and other immunoglobulin levels were within normal ranges. Consistent with this finding, Rasheed *et al*. reported a rare case of PA in a 19-year-old female without any underlying immunocompromised condition [14].

Pulmonary aspergilloma typically develops within pre-existing lung cavities [9]. Our patient had no history of chronic lung disease, tuberculosis, cystic fibrosis, or other cavitary disorders. However, histopathology revealed pulmonary infarction, abscess formation, and extensive tissue necrosis alongside dense fungal colonization. These findings point to an undiagnosed prior event, such as subclinical infection or focal ischemic injury as the most likely source of the cavity, which then provided a site for *A. flavus* growth. The patient worked as a carpenter and had long-term exposure to wood dust, which may have played a role. Wood dust can carry *Aspergillus* spores and has been linked to hypersensitivity pneumonitis and chronic airway inflammation [15]. Still, occupational exposure alone probably does not explain cavity formation in an otherwise healthy lung. Perhaps inhaling fungal spores over many years caused subtle lung damage, or helped the fungus settle once the cavity had formed.

Clinical presentation is variable, ranging from asymptomatic disease to severe respiratory manifestations. Our patient presented with persistent cough and dyspnea without hemoptysis, showing that pulmonary aspergilloma should also be considered in immunocompetent patients presenting with nonspecific respiratory symptoms.

Thoracic computed tomography plays an important role in the initial work-up of PA and typically shows a cavitary lesion with an intracavitary fungal mass [16]. However, radiological findings alone are insufficient for establishing the diagnosis, as the differential diagnosis includes pulmonary tuberculosis, hydatid cysts, lung abscesses, bronchogenic carcinoma, and Pneumocystis jirovecii pneumonia, all of which may present with similar radiological features [17]. Histopathological examination remains the gold standard for diagnosis, while microbiological evaluation, including direct microscopy and fungal culture of biopsy specimens, provides definitive confirmation [18], [19] In patients who are not surgical candidates, a presumptive diagnosis can be made based on characteristic CT findings combined with positive serum *Aspergillus* IgG [8]*. I*n our patient, the diagnosis was confirmed by histopathology, fungal culture, and β-tubulin gene sequencing.

The optimal management of PA remains controversial. Surgical resection is considered the treatment of choice for symptomatic aspergilloma because it removes both the fungal ball and the surrounding diseased lung tissue, despite its recognized operative morbidity [20], [21]. Systemic treatment with amphotericin B is inefficient and has a significant side-effect profile. Itraconazole remains the most extensively evaluated oral triazole for the management of pulmonary aspergillosis [22]. A multicenter clinical trial illustrated the efficacy of itraconazole at the daily dose of 200 mg for a duration of six months in treatment of aspergilloma, with a cure rate of 63.4% [23]. In a prospective study, Gupta *et al*. reported that patients treated with itraconazole, either at a fixed dose of 200 mg twice daily or using weight-based dosing, demonstrated a favorable clinical response, defined as control or reduction of hemoptysis, cough, dyspnea, and weight loss [24]. In our patient, segmental resection was successfully performed, followed by antifungal therapy. Although amphotericin B was initially administered *(as antifungal susceptibility testing had not yet been completed at the time treatment was initiated),* treatment was discontinued because of poor tolerance and severe tremors. Oral itraconazole was subsequently prescribed, resulting in complete clinical recovery. In addition, antifungal susceptibility testing performed according to the EUCAST method demonstrated the lowest MICs for posaconazole and anidulafungin, whereas amphotericin B and 5-fluorocytosine showed comparatively high MICs. Detailed susceptibility data for *A. flavus* pulmonary aspergilloma in immunocompetent patients remain limited and may contribute to more individualized antifungal therapy.

## Conclusion

We report a rare case of PA caused by *A. flavus* in a young immunocompetent patient with no prior lung disease. Definitive diagnosis required the integration of histopathology with molecular identification via β-tubulin gene sequencing. Antifungal susceptibility testing revealed revealed the lowest MICs for posaconazole, with higher values for amphotericin B and 5-fluorocytosine. The patient recovered completely after surgical resection combined with itraconazole. This report shows that PA should remain in the differential diagnosis of cavitary lung lesions even in immunocompetent hosts lacking traditional risk factors. This case also demonstrates how combing molecular identification, fungal culture, histopathology, and antifungal susceptibility testing can improve diagnostic precision and help antifungal treatment choices. While occupational wood dust exposure at work may have been a contributing factor, it likely occurred in combination with other underlying lung injuries that had not been recognized earlier.

## Ethics declaration

Written informed consent to take part in the study and to publish the article has been obtained from all participants or their legal representatives. The privacy rights of participants have been observed.

This study included organ or tissue donors. This study includes human biological material and consent was obtained by donors, or their next of kin or legal representatives, for use in this study and for publication of the article. The samples used in this research were not sourced from executed prisoners or prisoners of conscience.

This study was performed in compliance with relevant laws, regulatory frameworks and guidelines where the research took place. This study was approved by the Mashhad University of Medical Sciences. (Approval No. IR.MUMS. REC.1393.152).

This research follows the CARE guidelines and the CARE checklist.

## CRediT authorship contribution statement

**Mojtaba Taghizadeh Armaki:** Data curation. **Hossein Zarrinfar:** Writing – review & editing, Supervision. **Seyedeh Sabereh Mojtahedi:** Writing – original draft, Methodology.

## Consent

Written informed consent was obtained from the patient for publication of this case report and accompanying images. A copy of the written consent is available for review by the Editor-in-Chief of this journal on request.

## Ethics statement

This case (a part of the main project) was approved with an Ethics Committee code: IR.MUMS. REC.1393.152.

## Declaration of generative AI and AI-assisted technologies in the writing process

During the preparation of this work, the author(s) used ChatGPT in order to improve language clarity, grammar, and readability. After using this tool, the author(s) reviewed and edited the content as needed and take(s) full responsibility for the content of the publication.

## Declaration of Competing Interest

The authors declare that they have no known competing financial interests or personal relationships that could have appeared to influence the work reported in this paper.

## Data Availability

The data that support the findings of this study are available from the corresponding author upon reasonable request.

## References

[1] Akram W., Ejaz M.B., Mallhi T.H., Syed Sulaiman S.A.B., Khan A.H. (2021). Clinical manifestations, associated risk factors and treatment outcomes of Chronic Pulmonary Aspergillosis (CPA): Experiences from a tertiary care hospital in Lahore, Pakistan. PLoS One.

[2] Hosseinikargar N., Basiri R., Asadzadeh M., Najafzadeh M.J., Zarrinfar H. (2021). First report of invasive Aspergillus rhinosinusitis in a critically ill COVID-19 patient affected by acute myeloid leukemia, northeastern Iran. Clin case Rep.

[3] Aliabadi M.M., Sarasyabi M.S., Asoudeh R., Mostafavi K., Emadzadeh M., Zarrinfar H. (2026). Allergic fungal rhinosinusitis and its fungal species in Eastern Mediterranean: A systematic review. J Med Mycol.

[4] Luo W., Guo Q., Luo W., Chu M., Su Y., Zhang X. (2025). Embolization for Hemoptysis in Pulmonary Aspergillosis: Effectiveness and Risk Factors for Recurrence. J Vasc Interv Radiol.

[5] Teng G.L., Huang Q., Xu L., Chi J.Y., Wang C., Hu H. (2022). Clinical features and risk factors of pulmonary tuberculosis complicated with pulmonary aspergillosis. Eur Rev Med Pharmacol Sci.

[6] Zarrinfar H., Mirhendi H., Fata A., Khodadadi H., Kordbacheh P. (2015). Detection of Aspergillus flavusand A. fumigatus in Bronchoalveolar Lavage Specimens of Hematopoietic Stem Cell Transplants and Hematological Malignancies Patients by Real-Time Polymerase Chain Reaction, Nested PCR and Mycological Assays. Jundishapur J Microbiol.

[7] DAI Z., ZHAO H., CAI S., LV Y., TONG W. (2013). Invasive pulmonary aspergillosis in non-neutropenic patients with and without underlying disease: A single-centre retrospective analysis of 52 subjects. Respirology.

[8] Chakraborty R.K., Baradhi K.M. Aspergilloma. StatPearls. Treasure Island (FL): StatPearls Publishing Copyright © 2022, StatPearls Publishing LLC.; 2022.

[9] Mikilps-Mikgelbs R., Aksjutins F., Aleksejeva E., Bināns H., Coelho B.L., Gaidukova M. (2026). Minimally invasive management of a centrally located pulmonary aspergilloma in an adolescent patient. Multimed Man Cardiothorac Surg MMCTS.

[10] Society C.T. Clinical practice guidelines for the diagnosis and management of invasive pulmonary fungal diseases (2025 Edition). Zhonghua jie he he hu xi za zhi= Zhonghua jiehe he huxi zazhi= Chinese journal of tuberculosis and respiratory diseases. 2025;48(12):1104-26. 10.3760/cma.j.cn112147-20250819-00501 41362140

[11] Ranjan N., Ishak A., Cherabuddi M., Garrett S., Sheth R., Wadhwa A. (2025). Massive hemoptysis due to pulmonary artery pseudoaneurysm in invasive aspergillosis in a patient with interstitial lung disease and suspected Aspergillioma: A case report. Respir Med Case Rep.

[12] (2008). EUCAST Technical Note on the method for the determination of broth dilution minimum inhibitory concentrations of antifungal agents for conidia-forming moulds. Clin Microbiol Infect.

[13] Seo C., Dumoulin E., Thornton C.S. (2026). Spore Wars: A Comprehensive Review of Pulmonary Aspergilloma and Its Clinical Management. Chest.

[14] Rasheed A., McCloskey A., Foroutan S., Waheed A., Rodgers A., Seraj S.M. (2022). Pulmonary Aspergilloma in a Young Immunocompetent Female: A Rare Clinical Dilemma. Cureus.

[15] Abateneh G., Gizaw Z., Gebrehiwot M., Worede E.A. (2024). Prevalence of chronic respiratory symptoms and associated factors among woodwork workers in Bahir Dar City, Ethiopia; a comparative cross-sectional study. BMC Pulm Med.

[16] Ngu S., Narula N., Abureesh M., Li J.J., Chalhoub M. (2020). Endobronchial aspergilloma—a comprehensive literature review with focus on diagnosis and treatment modalities. Eur J Clin Microbiol & Infect Dis.

[17] Franquet T., Müller N.L., Giménez A., Guembe P., de La Torre J., Bagué S. (2001). Spectrum of pulmonary aspergillosis: histologic, clinical, and radiologic findings. Radiographics.

[18] Schelenz S., Barnes R.A., Barton R.C., Cleverley J.R., Lucas S.B., Kibbler C.C. (2015). British Society for Medical Mycology best practice recommendations for the diagnosis of serious fungal diseases. Lancet Infect Dis.

[19] Guziejko K., Klukowska K., Budzińska U., Mróz R.M. (2021). Case Report: Chronic Pulmonary Aspergillosis-An Unusual Long-Term Complication of Lung Cancer Treatment. Front Med (Lausanne).

[20] Jewkes J., Kay P.H., Paneth M., Citron K.M. (1983). Pulmonary aspergilloma: analysis of prognosis in relation to haemoptysis and survey of treatment. Thorax.

[21] Liebler J.M., Markin C.J. (2000). Fiberoptic bronchoscopy for diagnosis and treatment. Crit care Clin.

[22] Zaragoza R., Maseda E., Pemán J. (2021). Tratamiento antifúngico individualizado en el paciente crítico con infección fúngica invasora. Rev Iberoam De Micolía.

[23] Tsubura E. (1997). Multicenter clinical trial of itraconazole in the treatment of pulmonary aspergilloma. Pulmonary Aspergilloma Study Group. Kekkaku.

[24] Gupta P.R., Jain S., Kewlani J.P. (2015). A comparative study of itraconazole in various dose schedules in the treatment of pulmonary aspergilloma in treated patients of pulmonary tuberculosis. Lung India.

